# Peripheral Blood T-Cell Fitness Is Diminished in Patients With Pancreatic Carcinoma but Can Be Improved With Homeostatic Cytokines

**DOI:** 10.1016/j.jcmgh.2019.07.008

**Published:** 2019-08-06

**Authors:** J. Xu, H. Sai, Y. Li, A.C. Jordan, S.E. McGettigan, J.-H. Chen, F. Bedoya, J.A. Fraietta, W.L. Gladney, J.Joseph Melenhorst, G.L. Beatty

**Affiliations:** 1 Center for Cellular Immunotherapies, Perelman School of Medicine at the University of Pennsylvania, Philadelphia, Pennsylvania; 2 Department of Pathology and Laboratory Medicine, Perelman School of Medicine at the University of Pennsylvania, Philadelphia, Pennsylvania; 4 Division of Hematology-Oncology, Department of Medicine, Perelman School of Medicine at the University of Pennsylvania, Philadelphia, Pennsylvania; 5 Department of Microbiology, Perelman School of Medicine at the University of Pennsylvania, Philadelphia, Pennsylvania; 3 Abramson Cancer Center, University of Pennsylvania, Philadelphia, Pennsylvania

**Keywords:** CAR, chimeric antigen receptor, CCR, C-C chemokine receptor, DEG, differentially expressed gene, IL, interleukin, LAG3, lymphocyte-activation gene 3, PCR, polymerase chain reaction, PDAC, pancreatic ductal adenocarcinoma, PD1, programmed cell death protein 1

Pancreatic ductal adenocarcinoma (PDAC) shows remarkable resistance to immunotherapy.^1^ Although cancer cell–intrinsic mechanisms are known to support immune escape, T-cell “fitness” also has emerged as a key determinant of immunotherapy outcomes.^2^ In PDAC, adoptively transferred T cells show limited expansion after infusion.^3^ Moreover, more than 50% of PDAC patients fail to mount productive T-cell responses to tumor vaccines.^4^ T-cell hypofunction in PDAC, however, remains ill defined. Here, we show that chemotherapy-refractory PDAC patients harbor increased frequencies of terminally differentiated effector, rather than exhausted, peripheral blood T cells, that show an altered transcriptional profile with decreased functionality.

We examined the proliferative capacity of T cells isolated from the blood of chemotherapy-refractory PDAC patients compared with healthy volunteers (Supplementary Figure 1*A* and *B*). We studied this patient subset because they represent a major population evaluated in immunotherapy trials and, to date, responses have been exceptionally poor.^1^ We found that T-cell subset frequencies were similar between patients and volunteers (Supplementary Figure 1*C*). However, patient-derived T cells showed significantly decreased proliferative capacity (Figure 1*A*) that was independent of age (Supplementary Figure 1*D*). This dysfunction was a result of decreased proliferation by effector memory and effector CD8^+^ and CD4^+^ T cells (Supplementary Figure 1*E*). In contrast, proliferation by naive-like and central memory T-cell subsets were similar (Supplementary Figure 1*E*). The frequency of naive-like T cells was reduced in patients with a proportional increase in effector T cells (Supplementary Figure 1*F*). Patient-derived T cells also showed a decreased capacity to secrete interleukin (IL)6 and granulocyte-macrophage–colony-stimulating factor, but not other effector cytokines (eg, interferon-γ and tumor necrosis factor-α) (Supplementary Figure 2*A*). We did not examine for alterations in cytolytic function, which was a limitation of our analysis. Nonetheless, these data show diminished proliferative capacity by effector and effector memory peripheral blood T cells in chemotherapy-refractory PDAC patients.

**Figure 1:**
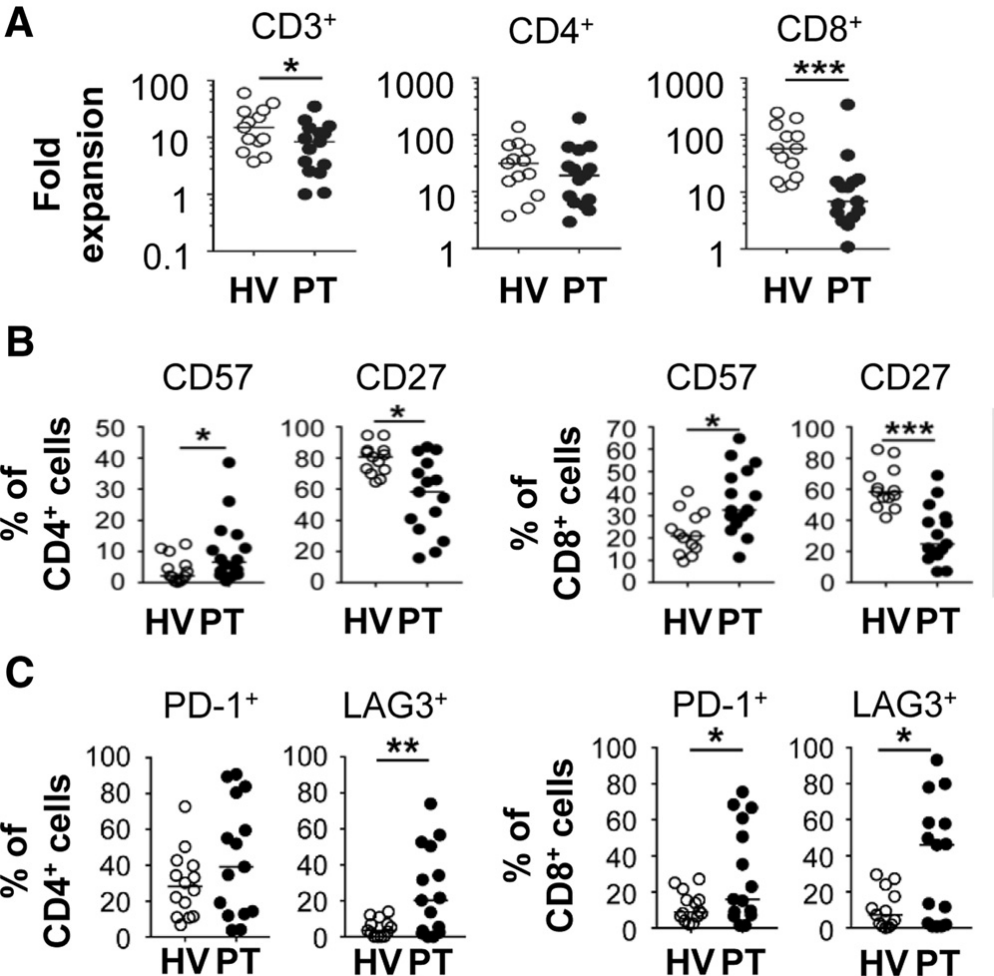
**(*A*) Fold expansion of T cells after polyclonal stimulation with anti-CD3/CD28 beads in IL2 for 10 days from healthy volunteers (HV) and PDAC patients (PT).** (*B*) Expression of molecules on unstimulated peripheral blood T cells. (*C*) Expression of immunoregulatory molecules on T cells after polyclonal stimulation. PT, *n* = 15; HV, *n* = 13.

T-cell differentiation is associated with distinct transcriptional profiles.^5^ From RNA sequencing of purified peripheral blood CD4^+^ and CD8^+^ T cells, we identified 310 and 955 differentially expressed genes (DEGs), respectively, between patients and volunteers with 155 DEGs shared across CD4^+^ and CD8^+^ T cells. Gene ontology analysis showed that DEGs down-regulated in patient CD8^+^ T cells were associated with cell proliferation and in patient CD4^+^ T cells were associated with apoptosis. Up-regulated genes in CD4^+^ T cells were associated with cell-cycle processes, and in CD8^+^ T cells were associated with cellular response to hypoxia and tumor necrosis factor–mediated signaling. Gene set enrichment analysis on genes up-regulated in CD8^+^ T cells showed enrichment associated with effector T cells (Supplementary Figure 2*B*). Based on this finding, we investigated genes involved in cellular differentiation and exhaustion. Surprisingly, we detected no association with T-cell exhaustion (eg, Eomesodermin [EOMES], PDCD1, and cytotoxic T-lymphocyte-associated protein 4 [CTLA-4]). Rather, patient-derived CD8^+^ T cells showed a decrease in cell survival genes (ie, B-cell lymphoma 2 [BCL2], IL7 receptor [IL7R]) and alterations in genes associated with effector activity including down-regulation of C-C chemokine receptor type 7 (CCR7) and up-regulation of interferon-gamma (IFNG), granzyme B (GZMB), granzyme A (GZMA), and Natural Killer Cell Receptor 2B4 (CD244) (Supplementary Figure 2*C*). Transcriptional changes in CD8^+^ T cells were confirmed by quantitative reverse-transcription polymerase chain reaction (Supplementary Figure 2*D*). Specifically, we detected down-regulation in RNA transcripts related to cellular signaling (salt inducible kinase 1 [SIK1]), differentiation (nucelar receptor related 1 protein [NR4A2], bZip Maf transcription factor [MAFF]), lymph node (A-kinase anchor protein 9 (AKAP9)) and bone marrow homing (C-X-C chemokine receptor type 4 [CXCR4]), and survival (IL7R).

We next profiled unstimulated peripheral blood T cells and found no significant differences in the expression of immunoregulatory molecules associated with T-cell exhaustion, including programmed cell death protein 1 (PD-1, or PDCD1), T-cell immunoglobulin and mucin-domain containing-3 (TIM3), and lymphocyte-activation gene 3 (LAG3) (Supplementary Figure 3*A*). We found no significant differences in natural or induced regulatory T-cell frequencies (Supplementary Figure 3*B*). In contrast, the frequency of HLA-DR, but not CD25, expressing CD4^+^ and CD8^+^ T cells was increased in patients (Supplementary Figure 3*C*). Patient-derived T cells also showed increased CD57 expression and loss of CD27 (Figure 1*B*), which is seen with T-cell senescence and terminal differentiation.^6^, ^7^, ^8^ In addition, patient-derived CD4^+^ and CD8^+^ T cells showed an increased propensity to express immunoregulatory molecules (including PD-1 and LAG3) after in vitro stimulation (Figure 1*C*, Supplementary Figure 3*D–F*).

Because T cells from patients showed a transcriptional profile consistent with decreased survival and lower levels of IL7R, we hypothesized that homeostatic growth factors may improve patient-derived T-cell function. We compared T cells activated in the presence of IL2 vs IL7/IL15, which support memory T-cell survival, proliferation, and recall responses.^9^, ^10^ We found a 2-fold increase in T-cell expansion ex vivo, with stimulation involving IL7/IL15 (Figure 2*A*). No difference in the expression of immunoregulatory molecules (PD1, LAG3, TIM3, and CD25) was observed after stimulation with IL2 compared with IL7/IL15 (Supplementary Figure 4*A*). However, CD4^+^ effector memory T cells, but not CD4^+^ or CD8^+^ effector T cells, showed increased expansion with IL7/IL15, implying that other factors regulate the decreased proliferative capacity of effector T cells (Figure 2*B*, Supplementary Figure 4*B–D*).

**Figure 2:**
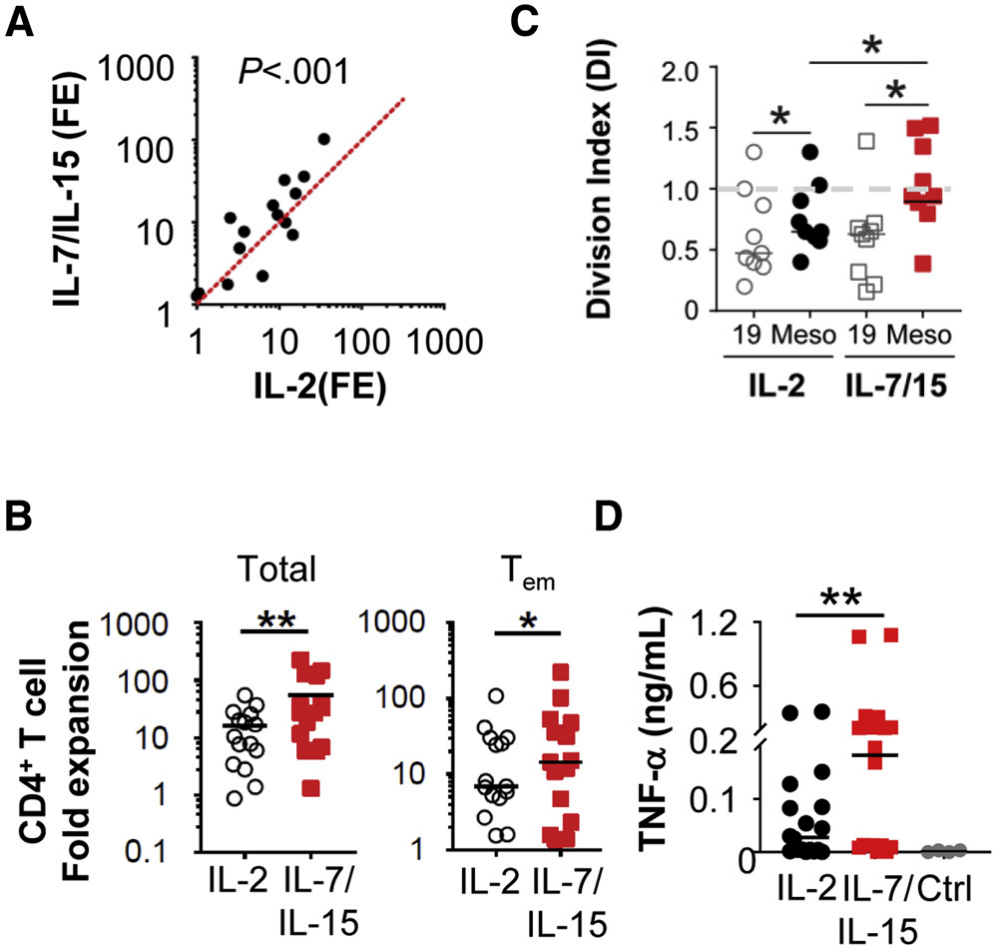
**(*A*) Fold expansion (FE) of polyclonally stimulated T cells from PDAC patients (*n* = 15) in the presence of IL2 or IL7/IL15.** (*B*) Fold expansion of CD4^+^ T-cell subsets. (*C*) Division index at day 5 and (*D*) cytokine release by mesothelin (Meso)-specific CAR T cells from patients (*n* = 9). TNF, tumor necrosis factor.

We tested the capacity of IL7/IL15 compared with IL2 to improve the functionality of patient-derived T cells responding to restimulation with a specific tumor antigen by introducing a mesothelin-specific chimeric antigen receptor (CAR) into expanded T cells. The transfection efficiency of the mesothelin CAR was 90%–98% after 24 hours. CAR T cells derived in the presence of IL2 contracted when restimulated with antigen and showed less cytokine production compared with IL7/IL15, which improved both the cytokine release capacity and expansion of CAR T cells (Figure 2*C* and *D*, Supplementary Figure 4*E*).

Together, our study shows that peripheral blood T cells in patients with chemotherapy-refractory PDAC harbor intrinsic alterations that limit their functionality, which may influence their potential to be harnessed for antitumor activity. This study offers insights into the defects in T-cell immunosurveillance associated with PDAC and suggests that strategies to reverse T-cell dysfunction may be necessary for advancing immunotherapy.

## Figures and Tables

**Supplementary Figure 1:**
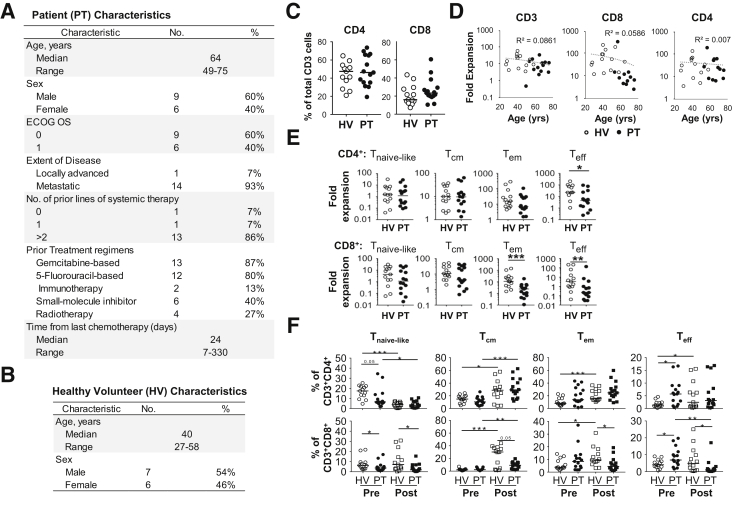
**Study participant characteristics for (*A*) patients (PT) with pancreatic ductal adenocarcinoma and (*B*) healthy volunteers (HV).** (*C*) Shown are percentages of CD4^+^ and CD8^+^ cells among total live CD3^+^ T cells detected in peripheral blood mononuclear cells or elutriated lymphocytes collected from healthy volunteers (*n* = 13) and patients with PDAC (*n* = 15). (*D*) Relationship of age to fold expansion of T-cell subsets. The correlation coefficient is shown in the figure. (*E*) Fold expansion of T-cell subsets in IL2 for 10 days. (*F*) Frequency of T-cell subsets detected before and after expansion in vitro with anti-CD3/CD28 Dynabeads plus IL2 for 10 days. ECOG OS, Eastern Cooperative Oncology Group overall survival. ∗*P* < .05; ∗∗*P* < .01; ∗∗∗*P* < .001; ∗∗∗∗*P* < .0001.

**Supplementary Figure 2:**
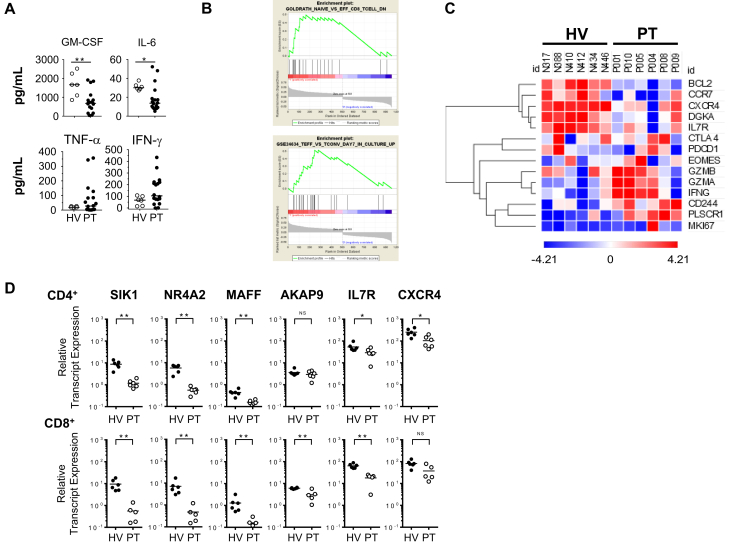
**(*A*) Cytokine secretion detected at 24 hours with T cells (patients [PT], *n* = 9) engineered to express a mesothelin-specific CAR and stimulated with mesothelin-expressing K562 cells.** (*B*) Gene set enrichment analysis of T-cell effector signaling genes in peripheral blood CD8^+^ T cells isolated from patients (*n* = 6) compared with healthy volunteers (HV) (*n* = 6). (*C*) Heat map of genes expressed by CD3^+^CD8^+^ T cells. (*D*) Messenger RNA levels of genes associated with differentiation, signaling, homing, and survival detected in sorted peripheral blood T cells as indicated. GM-CSF, granulocyte-macrophage–colony-stimulating factor. ∗*P* < .05; ∗∗*P* < .01; ∗∗∗*P* < .001; ∗∗∗∗*P* < .0001.

**Supplementary Figure 3:**
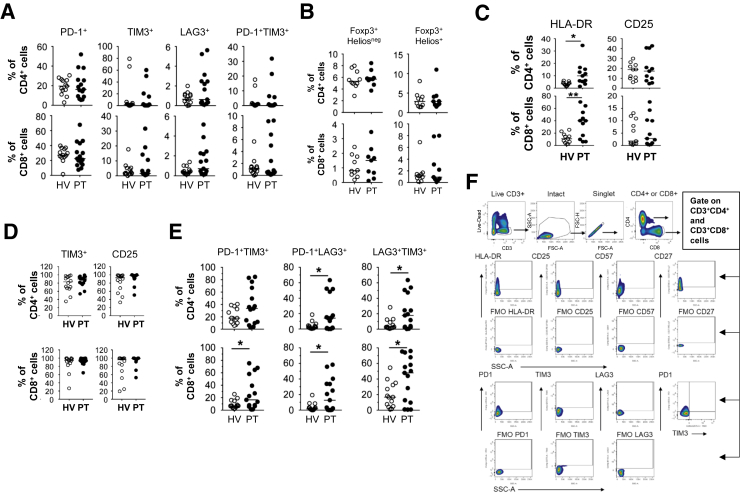
**(*A*) Immunoregulatory molecule expression on peripheral blood CD3**^**+**^**T-cell subsets from healthy volunteers (HV; *n* = 13) and patients (PT; *n* = 14).** (*B*) Percentages of induced (*left*) and natural (*right*) regulatory T-cell subsets among total CD4^+^ and CD8^+^ T cells (HV, *n* = 10; PT, *n* = 9). (*C*) Expression of molecules and (*D* and *E*) immunoregulatory molecules on CD3^+^ T-cell subsets after polyclonal stimulation with anti-CD3/CD28 Dynabeads in the presence of IL2 for 10 days (HV and PT, *n* = 11). (*F*) Flow cytometry gating strategy and fluorescence minus 1 controls. SSC-A, size scatter area. ∗*P* < .05; ∗∗*P* < .01; ∗∗∗*P* < .001; ∗∗∗∗*P* < .0001.

**Supplementary Figure 4:**
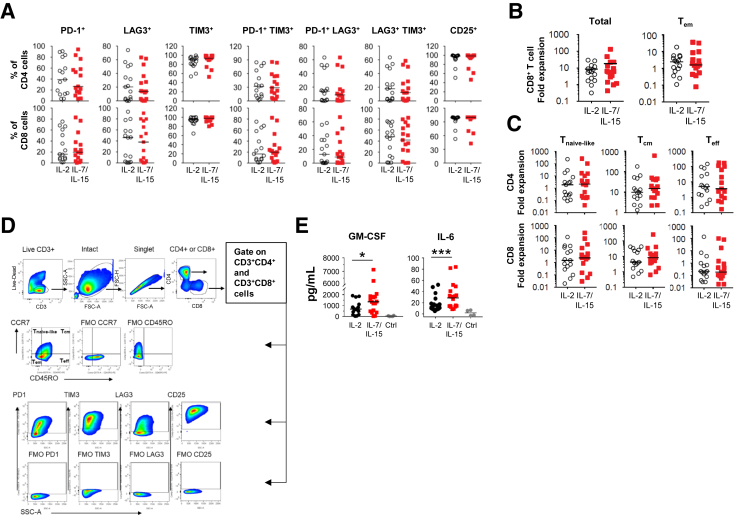
**(*A*) Percentages of CD4**^**+**^**and CD8**^**+**^**T cells (patients [PT], *n* = 14) expressing PD1, TIM3, LAG3, and CD25 after expansion in the presence of IL2 or IL7/IL15.** (*B*) Fold expansion of total and Teff CD8^+^ T-cell subsets in the presence of IL2 or IL7/IL15. Data for CD4^+^ T cells is shown in Figure 2. (*C*) Fold expansion of T naive-like, T central memory (Tcm), and T effector (Teff) CD3^+^ T-cell subsets in the presence of IL2 and IL7/IL15. (*D*) Flow cytometry gating strategy and fluorescence minus 1 controls. (*E*) Cytokine release by mesothelin (Meso)-specific CAR T cells from patients. Data for TNF-α is shown in Figure 2. Ctrl, control; GM-CSF, granulocyte-macrophage–colony-stimulating factor; SSC-A, size scatter area. ∗*P* < .05; ∗∗*P* < .01; ∗∗∗*P* < .001; ∗∗∗∗*P* < .0001.

## Supplementary Materials and Methods

### Clinical Samples

Patients with advanced pancreatic ductal adenocarcinoma (*n* = 15) and healthy volunteers (*n* = 13) were enrolled at the Abramson Cancer Center at the University of Pennsylvania (Philadelphia, PA). All patient samples were obtained after written informed consent was obtained. Peripheral blood samples were collected by elutriation or Ficoll centrifugation and cryopreserved in liquid nitrogen until analysis. The study was approved by the institutional review board of the University of Pennsylvania and conducted in accordance with the 1996 Declaration of Helsinki.

### Ex Vivo Expansion of T Cells

Cryopreserved elutriated lymphocytes or Ficoll-separated peripheral blood mononuclear cells were thawed, and CD3^+^ T cells were purified by negative selection using the Pan T selection kit (Miltenyi Biotec, Bergisch Gladbach, Germany). The purity of isolated CD3^+^ cells was 70%–90% (data not shown). Enriched CD3^+^ T cells were stimulated with CTS Dynabeads CD3/CD28 (Thermo Fisher Scientific, Waltham, MA) at a cell-to-bead ratio of 1:3, in media^1^ with 100 U/mL recombinant human IL2 (Prometheus Laboratories, San Diego, CA) or with 5 ng/mL recombinant human IL7 and IL15 (Miltenyi Biotec) for 10 days. Modified medium was prepared as previously described.^1^ Cell number and size were monitored by the Multisizer 4 (Beckman, Brea, CA) or Luna automated cell counter (Logos Biosystems, Anyang-si, Gyeonggi-do, South Korea), and viability was assessed by trypan blue staining every 2–3 days. The initial T-cell concentration was 1 × 10^6^ cells per milliliter. After 10 days, beads were removed from expanded T cells and cells were analyzed by fluorescence-activated cell sorter staining or cryopreserved. Cells were cryopreserved in medium containing 70% X-VIVO15 (Lonza, Basel, Switzerland), 10% dimethyl sulfoxide (Thermo Fisher Scientific), and 20% human serum (Sigma-Aldrich, St. Louis, MO). Fold expansion was calculated as follows: cell number on day 10 divided by cell number on day 0.

### Flow Cytometry Analysis

T cells were stained with fixable live/dead Violet or Aqua dye (Thermo Fisher Scientific), and stained with commercially available antihuman antibodies. T-cell subsets were defined as follows: (1) naive T cells: CCR7^+^CD45RO^neg^CD27^+^CD127^+^CD95^neg^; (2) stem-cell memory: CCR7^+^CD45RO^neg^CD27^+^CD127^+^CD95^+^; (3) central memory T cells: CCR7^+^CD45RO^+^; (4) effector memory T cells: CCR7^neg^CD45RO^+^; and (5) effector T cells: CCR7^neg^CD45RO^neg^.

Mesothelin ss1 CAR expression was detected after electroporation with messenger RNA mesothelin ss1 CAR (by biotin-SP(long spacer) affiniPure goat-anti-mouse IgG; Jackson ImmunoResearch (West Grove, PA); and streptavidin-phycoerythrin (PE) as secondary antibody; BD, Franklin Lakes, NJ). Fluorescence-activated cell sorter buffer was prepared with phosphate-buffered saline containing 3% fetal bovine serum (Gemini Bio-Products, West Sacramento, CA) and 0.05% sodium azide. Cells were fixed with 1% paraformaldehyde before analysis on a Fortessa flow cytometer (BD). Data were analyzed using FlowJo V10 (Tree Star, Ashland, OR).

### Purification of CD4^+^ and CD8^+^ T Cells

Cryopreserved cells from elutriated lymphocytes or cultured T cells were thawed and counted by a Luna automated cell counter. CD3^+^ T cells were isolated via the Pan T selection kit (Miltenyi Biotec). CD4^+^ or CD8^+^ T cells were enriched by positive selection microbeads (Miltenyi Biotec). Cell purity was routinely 80%–95%. Isolated CD4^+^ and CD8^+^ cells were subjected to TRIzol treatment for RNA isolation (Thermo Fisher Scientific).

### Quantitative Reverse Transcription Polymerase Chain Reaction

Total RNA from magnetic bead–sorted CD4^+^ and CD8^+^ T cells was extracted using the RNA Direct-zol MiniPrep Kit (Zymo Research, Irvine, CA). DNA fragments were generated by reverse-transcriptase polymerase chain reaction (Applied Biosystems, Foster City, CA). Real-time polymerase chain reaction was performed using the ViiA 7 real-time polymerase chain reaction system (Thermo Fisher Scientific).

### QuantSeq 3’ Messenger RNA Sequencing and Data Analysis

RNA was isolated from magnetic bead–sorted CD4^+^ and CD8^+^ T cells and submitted to the Genomics Facility at the Wistar Institute (Philadelphia, PA). RNA quality was assessed using 2100 Bioanalyzer (Agilent, Santa Clara, CA). Samples were prepared using a QuantSeq 3’ messenger RNA sequencing library preparation kit FWD for Illumina (Lexogen, Greenland, NH), and analyzed on a NextSeq 500 sequencing system (Illumina, San Diego, CA). FASTQ files were uploaded to BaseSpace Suite (Illumina) and aligned using the RNA sequencing alignment application (version 1.0.0). Output files were analyzed using DESeq2 to determine differentially expressed genes and generate an expression heatmap using a false-discovery rate of 0.1 and an adjusted cut-off *P* value of .05. Gene ontology of differentially expressed genes was determined using the protein analysis through evolutionary relationships (PANTHER) application. Gene-set enrichment analysis (version 3.0)^2^ was used to determine biological processes enriched in T cells from patients compared with normal donors.

### Generation of Mesothelin ss1 CAR Messenger RNA by In Vitro Transcription

The anti–mesothelin ss1 scFv CAR messenger RNA construct contains tumor necrosis factor receptor super family member 9 (TNFRSF9, or 4-1BB) and T-cell receptor (TCR)-ζ signaling domains.^3^ In vitro transcription RNA for mesothelin ss1 CAR was prepared using the T7 mScript messenger RNA production system (Cellscript, Madison, WI).^3^

### Mesothelin ss1 CAR Messenger RNA Electroporation of Human T Cells

Cryopreserved T cells were thawed and rested overnight. T cells were electroporated with 1 μg RNA in vitro transcription mesothelin ss1 CAR RNA per 10^6^ cells using a BTX ECM830 (Harvard Apparatus BTX, Holliston, MA) electroporation machine. After 24 hours, mesothelin ss1 CAR expression was detected by flow cytometry. Transfection efficiency was routinely 90%–98%.

### T-Cell Proliferation by Restimulation With Target Cells

Mesothelin ss1 CAR messenger RNA electroporated T cells were labeled with 1 μmol/mL Carboxyfluorescein succinimidyl ester (CFSE) (Thermo Fisher Scientific) and co-cultured in cytokine-free modified media for 5 days with pre-irradiated K562 cells expressing target human mesothelin or nontarget human CD19. K562 cells were cultured using filtered (0.22 μm) complete media containing RPMI 1640 supplemented with 10% heat-inactivated fetal bovine serum, 100 U/mL penicillin, and 100 μg/mL streptomycin (Thermo Fisher Scientific and Germini Bio-products). K562 cell lines were transduced with lentivirus expressing human mesothelin or CD19 via pTRPE or pELPS lentiviral vector, sorted, and expanded in culture. Ectopic expression of mesothelin or CD19 was confirmed by flow cytometry. Cell-line authentication was performed by the University of Arizona Genetics Core, on the basis of criteria established by the International Cell Line Authentication Committee. Short-tandem-repeat profiling revealed that these cell lines were above the 80% match threshold. K562 cells were irradiated using XRAD 320iX (Precision X-ray, Branford, CT) at a dose of 100 Gy. T cells and K562 cell lines were seeded at 50,000 cells per well at a T:K562 ratio of 1:1. Twenty-four hours after co-culture, supernatants were collected for Luminex analysis (Luminex, Austin, TX). Five days later, T-cell proliferation was assessed by flow cytometry. Division index of gated live CD3 cells was determined based on CFSE dilution (FlowJo version 9.9.3).

### Cytokine Analysis Using Luminex

Twenty-four–hour supernatants were collected for Luminex analysis using the human Th17 magnetic bead panel Premixed-25 Plex (EMD Millipore, Burlington, MA). Specific antigen-mediated cytokine release is normalized as cytokine release (T+K562-Meso) minus cytokine release (T+K562-CD19).

### Statistical Analysis

Statistical significance was determined using Prism (GraphPad, San Diego, CA). Group comparison testing was performed using an unpaired or paired Student *t* test, the Mann–Whitney *U* test, or the Wilcoxon matched-pairs signed-rank test. For multiple comparisons, 1-way analysis of variance was used, followed by the Bonferroni post-test. **P* < .05; ***P* < .01; ****P* < .001; *****P* < .0001.

### Data Availability

The authors declare that the data supporting the findings of this study are available within this article and its Supplementary files.
